# Epigenetic Signatures of Cell States in Aging

**DOI:** 10.1093/geroni/igaa057.434

**Published:** 2020-12-16

**Authors:** Diana Leung, Morgan Levine

**Affiliations:** 1 Yale University, Elmhurst, New York, United States; 2 Yale University School of Medicine, New Haven, Connecticut, United States

## Abstract

Epigenetic clocks based on DNA methylation (DNAm) show striking age correlations and predict various outcomes. Patterns of DNAm also reflect critical mechanisms in differentiation and proliferation. As such, an outstanding question is whether part of the signal epigenetic clocks are capturing represent shifts in the proportions of somatic stem cells, senescence cells, and/or tumorigenic cells. Here, we assembled various methylation datasets that captured relevant phenomena, including pluripotent stem cells, differentiation, senescence, and cancer, and performed weighted network analysis to cluster and compare DNAm modules. We find overlapping clusters between in vitro samples and in vivo tissue samples, suggesting that cell-level phenomena like cell replication, senescence, and cancer intersect with age-related epigenetic signatures. While the effects of aging manifest at multiple systems levels, from the genome to clinical phenotypes, these analyses may help provide insight to the contribution of cell phenotype dynamics to the general aging phenomenon.

